# Perilymphatic hyperension

**DOI:** 10.1016/S1808-8694(15)30983-6

**Published:** 2015-10-19

**Authors:** João Alcides Miranda, Fábio Akira Suzuki, Marcello Henrique de Carvalho Borges

**Affiliations:** a Resident of the Otorhinolaryngology Unit of the Sao Francisco University Hospital, Bragança Paulista, SP; b Doctor in Otorhinolaryngology graduated at UNIFESP - EPM, Head of the Otorhinolaryngology Unit of the Sao Francisco University Hospital, Bragança Paulista, SP, Vice-coordinator of the post-graduate course in Otorhinolaryngology of the Servidor Publico Estadual Hospital (HSPE); c Resident of the Otorhinolaryngology Unit of the Sao Francisco University Hospital, Bragança Paulista, SP. This study was done in the Sao Francisco University Hospital, Bragança Paulista

**Keywords:** stapedectomy, gusher, otosclerosis

## INTRODUCTION

Gusher or perilymphatic fistula is a complication seen during stapedectomy or stapedotomy. It is characterized by sudden perilymphorrhage immediately after platinotomy^1^, ^2^, ^3^. It is a rare entity, arising in 1 of every 1,000 operated cases^2^, ^4^. There is currently no agreement among authors about the best strategy to treat gusher^5^. Interruption of the surgical procedure is usually recommended^1^, ^2^, ^6^.

## CASE REPORT

A female 38 year old patient complained of mostly left hypoacusis for the past 4 years; mild bilateral tympanic membrane retraction was present. Audiometry disclosed moderate left conduction loss and slight right conduction loss; immitance testing revealed an A curve and absence of stapedian reflexes bilaterally. The diagnosis was otosclerosis and left stapedectomy was recommended. Perilymph gushed out during microperforation of the platina of the stapes (gusher). The procedure was interrupted and fat from the left auricle was used for orifice tamponing, which stopped lymphorrhage.

## DISCUSSION

This complication of stapedotomy is an unexpected event and there are no warning signs that could alert the surgeon^1^, ^4^. This report describes a case of perilymphatic fistula in which we decided for interruption of the surgical procedure and tamponade of the oval window with fat, in agreement with Cassano et al.^1^, Rocha et al.^2^ and Hungria^6^. Couvreur et al.^4^, in a study of 4 patients that had intraoperative perilymphatic fistulae in which surgery was interrupted, mention the possibility of continuing the procedure in favorable cases where the orifice for inserting the prosthesis has not been excessively widened. In our case report post-operative audiometry detected mild worsening of hearing compared to pre-operative audiometry. Therefore, we opted for regular outpatient monitoring.

## FINAL COMMENTS

Gusher is a rare complication of otological surgery. When present, it becomes difficult to complete the surgical procedure; no true benefits have been observed to justify continuing the operation.
